# Factor VIII companion diagnostic for haemophilia

**DOI:** 10.3389/fbioe.2022.1006600

**Published:** 2022-10-05

**Authors:** Chunxiao Hu, Valerio F. Annese, Christos Giagkoulovits, Michael P. Barrett, David R. S. Cumming

**Affiliations:** ^1^ Division of Electronics and Nanoscale Engineering, School of Engineering, University of Glasgow, Glasgow, United Kingdom; ^2^ Wellcome Centre for Molecular Parasitology, Institute of Infection, Immunity, and Inflammation, University of Glasgow, Glasgow, United Kingdom

**Keywords:** diagnostic, point-of-care, haemophilia, factor VIII, chromogenic, photodiode

## Abstract

Haemophilia is predominantly an inherited disorder that impairs the body’s ability to make blood clots, a process needed to stop bleeding. The condition of this disease is complex to manage, but many patients do so through home therapy and often only see their core multidisciplinary healthcare team annually. There is an increasing need for patients to be able to monitor their condition efficiently at home while staying connected with their healthcare team. As a consequence, a low-cost handheld self-monitoring solution for clotting factor is required. Here we have demonstrated a suitable one-step Factor VIII companion diagnostic sensing approach based on a chromogenic assay for haemophilia A. The results show comparable performance to the gold standard method. Our approach is able to deliver accurate cost-effective results in under 5 min from undiluted human plasma. It has the potential to be able to reduce the human and monetary costs of over- or under-medication for haemophiliacs.

## Introduction

Haemophilia is a mostly inherited genetic disorder that impairs the body’s ability to make blood clots, a process needed to stop bleeding (Dameshek, 1954; Green, 2016). There are two main types of haemophilia (Bowen, 2002): haemophilia A, which occurs because of low amounts of clotting factor VIII, and haemophilia B, which occurs because of low levels of clotting factor IX. Globally, the prevalence of haemophilia A is around one in 5,000 male births, and one in 30 ,000 male births for haemophilia B (Rosendaal and Briet, 1990). In this work we focus on Haemophilia A that is the dominant type. The lower the amount of the relevant factor, the more likely it is that bleeding will occur that can lead to serious health problems. Individuals with less than 1% active factor are classified as having severe haemophilia and are required to be given clotting factors preventively 2 to 3 times per week (Lindvall et al., 2006; Peyvandi et al., 2016) resulting in average costs of $300,000 per patient per year (Sharma et al., 2020). Haemophilia affects around 1.125 million people world-wide according to the data shown in 2019 (Iorio et al., 2019). Individuals classified as having severe haemophilia represent approximately one-third of the haemophilia population (O'Hara et al., 2017). In addition to the distress caused to patients, haemophilia is therefore a considerable burden of both labour and cost to healthcare services.

Conventional methods used for monitoring the clotting factors include the 1-Stage PT-Based assay, 1-Stage APTT-Based assay, and 2-Stage APTT/Chromogenic assay (PT: prothrombin time; APTT: activated partial thromboplastin time) (Lossing et al., 1977; Rosen, 1984b; Duncan et al., 1985; Yuan et al., 2007). The general principles of all functional clotting factor assays are the same and involve plotting the clotting times against sample dilution. The degree of correction of the clotting time, when the plasma is added to a clotting system specifically deficient in the clotting factor to be measured, allows the level of that clotting factor to be determined (Duncan et al., 1985). All of these assays require a skilled person to perform with diluted plasma using a benchtop setup that is time-consuming and costly.

Point of Care (PoC) technology has been widely used for helping the diagnostics or monitoring of diseases such as diabetes (Blake and Nathan, 2004; Motta et al., 2017) and cancers (Hayes et al., 2018; Sandbhor Gaikwad and Banerjee, 2018). In recent years, there is an increasing trend towards PoC and home use diagnostics to aid the management of complex chronic diseases. This “self-care” model is well established for diseases such as diabetes; the management of other chronic diseases would also benefit from such regular monitoring (Bodenheimer et al., 2002; Yager et al., 2008; Shrivastava et al., 2013). The condition of haemophilia A for any individual is complex to manage (overdosing of clotting factor medicines leads to clots/stroke and underdosing cannot stop bleeding) (Hazendonk et al., 2015), but many patients do so through home therapy and often only see their core multidisciplinary healthcare team once a year. There is therefore an increasing need for patients to be able to monitor their condition more efficiently at home while staying connected with their healthcare team. A low cost handheld self-monitoring device for clotting factor would greatly support patient treatment.

In this paper, we have demonstrated an innovated one-step chromogenic approach for rapid measurement of the Factor VIII activity levels on a mobile, digital, low cost handheld self-monitoring sensing platform. The system is underpinned by the conventional chromogenic assay, but we have introduced methods to remove complicated operating steps: no dilution or defibrination of the sample is required, no incubation steps are involved, and minimal training is required for the operator. The colour change generated from the chromogenic assay is measured and recorded by the photodiode sensing platform in real-time. It is designed to allow the rapid and simultaneous measurement of multiple coagulation factors of interest (e.g., Factor VIII and Thrombin) from a single drop of capillary finger-stick blood or plasma. The sensing platform is based on CMOS technology, which has the advantages of low-cost, portable, and stable.

We have demonstrated the performance of the one-step chromogenic approach with Factor VIII test in a prototype form. Our results show comparable result to the gold standard method, but with less sample volume, simpler operation, and quicker response, which makes our sensing system a potential candidate for Factor VIII companion diagnosis by haemophilia A patients at home.

## Materials and methods

In this work we demonstrate a novel assay method that we implement on a silicon chip-based sensor.

### Conventional chromogenic assay

The chromogenic Factor VIII activity assay has been widely used by researchers and clinicians and in some cases it might be preferable to clot-based assays (Moser and Funk, 2014). It is used in some specialized haemophilia reference centres and is recommended for the diagnosis of mild haemophilia A since this assay is considered to better reflect the severity status of haemophilia patients than the conventional clot-based assay (Rodgers and Duncan, 2017; Al Moosawi et al., 2019).

**FIGURE 1:**
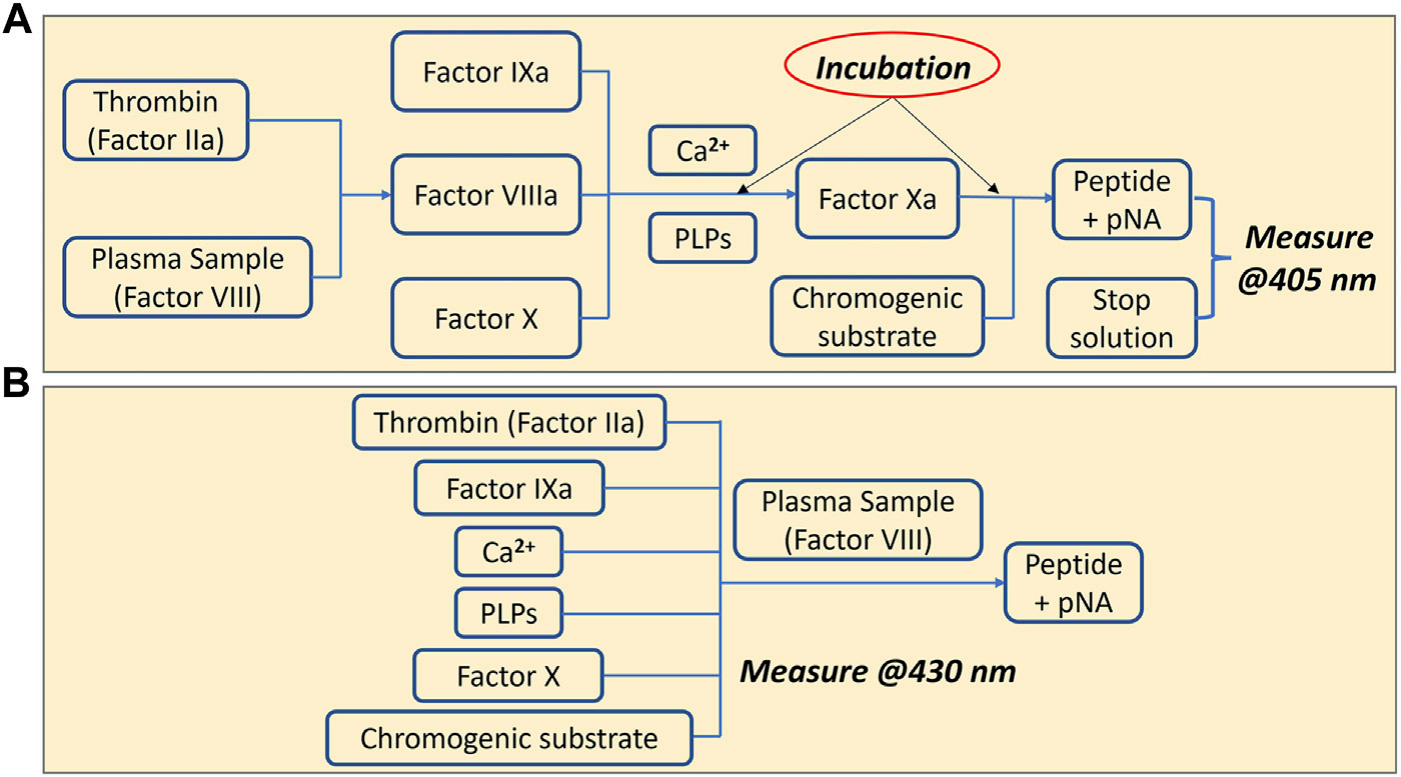
Chromogenic assay for measuring Factor VIII. **(A)** Conventional chromogenic method (Factor VIIIa, Factor IXa, and Factor Xa are the activated form of each factor) **(B)** One-step chromogenic approach on our sensing platform.

**FIGURE 2:**
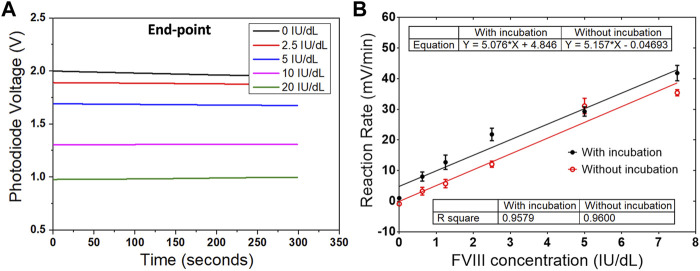
Results from both conventional and one-step chromogenic assay. **(A)** Reaction curves from the conventional assay, which record the end-point values from five different concentrations of Factor VIII. **(B)** Comparison of the reaction rates between the measurements with and without the incubation step. Data are the mean ± SEM with three repeated measurements.

The conventional chromogenic assay (Rosen, 1984a) for detecting Factor VIII involves an incubation step to generate Factor Xa and a second stage to determine the amount of Factor Xa produced. The working principle is shown in Figure 1A. In the presence of a constant amount of Factor IXa, phospholipids (PLPs) and calcium (Ca^2+^), thrombin activated Factor VIII forms an enzymatic complex that activates Factor X supplied in the assay at a constant concentration, and in excess, to Factor Xa. This activity is directly related to the amount of Factor VIII, which is the limiting factor in the presence of a constant and in excess amount of Factor IXa. The Factor Xa that is generated is then measured by its activity on a specific Factor Xa chromogenic substrate (see Chemical Preparation below). Factor Xa cleaves the substrate and releases *p-nitroaniline* (pNA). The amount of pNA generated is directly proportional to the Factor Xa activity. The absorbance of pNA is measured and since the colour intensity produced is directly proportional to the amount of Factor Xa, which in turn is directly proportional to the amount of Factor VIII, the Factor VIII levels may be calculated from the absorbance of the sample at the operating wavelength. The conventional assay contains at least two incubation steps and each takes 5 min at 37 C. All the samples and reagents are required to be preincubated at 37°C prior to the assay. The assay also includes a standard plasma dilution of 1:40 or 1:20. The end point value is recorded (Figure 2A).

### One-step chromogenic approach

To simplify the procedure of the assay, a one-step chromogenic approach was adapted on to the described CMOS sensing platform, based on the modification of the conventional method (Figure 1B). The objective is to remove all the incubation steps and to achieve one-step operation that requires only the addition of the undiluted plasma sample. To achieve that, all the reagents required for the assay except the plasma containing Factor VIII were mixed and placed on the chip surface of the sensing platform prior to the measurement. At this stage, without Factor VIII, no reaction occurs. Different to the conventional method, no thrombin inhibitor was used, and additional high concentration of fibrin polymerization inhibitor was added to eliminate the generation of cross-linked fibrin polymer, which makes it possible to use the whole blood plasma with no dilution. The next step was to begin recording with the sensing platform before adding the plasma sample in order to provide a baseline signal. The plasma containing Factor VIII was then added to initiate the reaction, and the signal variation owing to the colour change was recorded and processed. The signal obtained is proportional to the level of Factor VIII in the sample. Compared to the conventional method which measures the end-point value, the reaction rate was calculated in this work based on a real-time measurement. Using this method, the assay time can be reduced from more than 20 min to under 5 min. Another advantage of measuring the reaction rate is the elimination of the incubation step. The same measurements were performed at two different temperatures. In one measurement, both the mixed solution with all the reagents except the Factor VIII and the plasma sample, were incubated at 37°C for 5 min prior to the assay. In a second measurement, all the components were prepared at room temperature. The reaction rates from both measurements were compared and no obvious difference was observed (Figure 2B). The same measurements were performed at two different temperatures. In one measurement, both the mixed solution with all the reagents except the Factor VIII and the plasma sample, were incubated at 37 C for 5 min prior to the assay (black curve in Figure 2B). In a second measurement, all the components were prepared at room temperature (red curve in Figure 2B). Both measurements have similar linearity and fitness according to the slope and *R* ^2^ value. In addition, since the measurement was recorded in real-time, a stop solution was not required.

### Silicon chip based sensing platform

**FIGURE 3:**
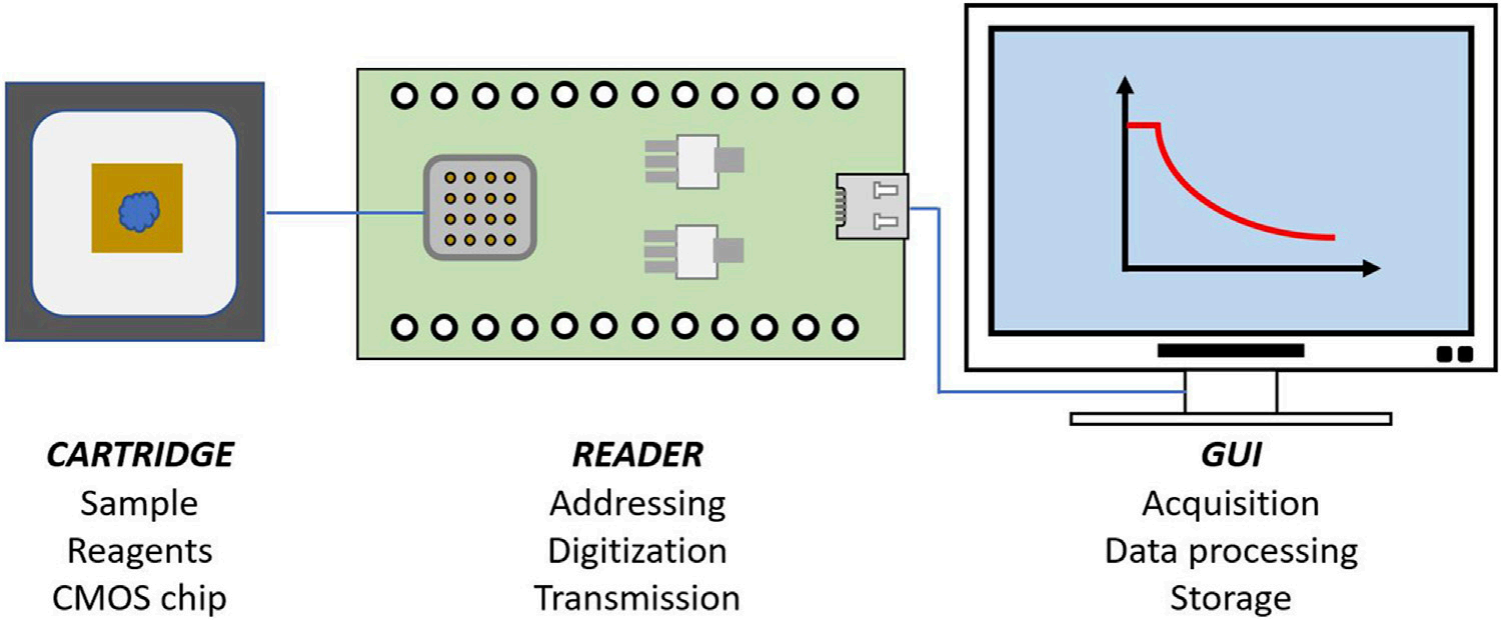
Illustration of the sensing platform. It combines a silicon (CMOS) based sensor chip on a disposable cartridge, a reader and a computing platform with GUI.

The entire sensor platform is a handheld device with cable connection to a computing platform (Figure 3) (Hu et al., 2018; Al-Rawhani et al., 2020; Annese et al., 2021). It is comprised of several components. The CMOS sensor chip, mounted on a chip carrier, has an array of 16 × 16 PD pixels; each PD pixel uses the well-established 3-transistor read-out circuit. The chip was fabricated using a commercially available CMOS 350 nm 4-metal process provided by austriaMicroSystems (AMS). A printed circuit board (PCB) was designed to interface the chip with an Arm mbed STM32 Nucleo-F334R8 board (STMicroelectronics, UK). The mbed microcontroller was programmed to provide addressing signals and to acquire the output readings from the PD array. The array can be used to exploit the statistical phenomenon of averaging signals from independent Gaussian noise sources, either over time or spatially, to improve the overall system sensitivity. The chip integrates addressing blocks to allow each sensor array to be controlled and operated independently or simultaneously as required. A LED mounted in a 3-D printed housing was used as a light source. The housing also doubled up as a light-proof unit to eliminate unwanted stray light from the measurements. The reaction and colour changes that occurred on the sensor chip were detected and recorded by the platform. The acquired data was transferred by universal serial bus (USB) to a computer running a Matlab program, where it was processed and analysed. The platform is interoperable with both types of computing device. The sensing wavelength for our photodiode is from 400 to 900 nm with peak at ∼ 600 nm. The chromogenic substrate pNA ranges from 300 to 480 nm with a peak at ∼ 405 nm. The intersection point between the two optical spectrums is 460 nm. Regarding the wavelength of the light source, 405 nm is the recommended wavelength for the chromogenic substrate, but silicon photodiodes are relatively insensitive at this wavelength. We experimented with three different wavelengths: 405, 430, and 450 nm, and found that 430 nm was the best choice for our measurements.

### Chemical preparation

The BIOPHEN™ FVIII:C chromogenic assay kit (221,402), BIOPHEN™ CS-11 (65)—Factor Xa Chromogenic Substrate (229,014), BIOPHEN™ Plasma Calibrator (222,101), and BIOPHEN™ Normal Control Plasma (223,201) were purchased from Quadratech Diagnostics. TEClot Factor VIII Deficient Plasma, Teco (P5301-010) was sourced from Alpha Laboratory. Fibrin polymerization inhibitor Gly-Pro-Arg-Pro amide (G5779), Human coagulation factor VIII concentrate (H0920000), Thrombin from human plasma (T6884), Calcium chloride (C1016), Trizma^®^ hydrochloride (T3253), Bovine Serum Albumin (A7030), sodium azide (S2002), and acetic acid (A6283) were bought from Sigma Aldrich.

The reaction buffer was prepared by adding 1% (w/v) bovine serum albumin to the Trizma^®^ hydrochloride (Tris-HCl) solution at pH 7.4, with the presence of 1 g/L sodium azide. 20% acetic acid was used as the stop solution.

In the one-step chromogenic approach, the initial step is to have all the reagents required for the assay except the plasma containing Factor VIII mixed and placed on the chip surface of the sensing platform. The mixed reagents include an excess amount of Human Factor X and Factor IXa. The mixture also contains human thrombin, phospholipids, fibrin polymerization inhibitor (0.5 mg/ml), calcium chloride and chromogenic substrate for Factor Xa (5 mg/ml). The reagents for which specific concentrations are not stated were taken from the BIOPHEN™ FVIII:C chromogenic assay kit.

### Data acquisition and analysis

The reader provides functionality for sensor multiplexing and addressing, data digitization and transmission to a personal computing device *via* a USB link. The reader is programmed before use with custom firmware. Data is digitized using the embedded 12-bit analogue to digital converter with an average rate of 36 frames per second.

As mentioned previously, instead of end-point value, the reaction rate of the assay was calculated and used to determine the Factor VIII level in the plasma sample. The point when the plasma sample was added was counted as time *t* = 0, and the signal change due to the reaction was recorded in real-time. The change of the signal with respect to time in a fixed interval gave the reaction rate, which was calculated by dividing the magnitude of the signal change by the corresponding time. The limit of detection (LOD) was quantified using the “International Union of Pure and Applied Chemistry” (IUPAC) definition. The average (μc) and standard deviation (δc) of the initial reaction rate for negative controls (common to all the assays) were calculated and consequently, the LOD (μc + 3.3·δc) was obtained.

## Results

### Result from diluted plasma

**FIGURE 4:**
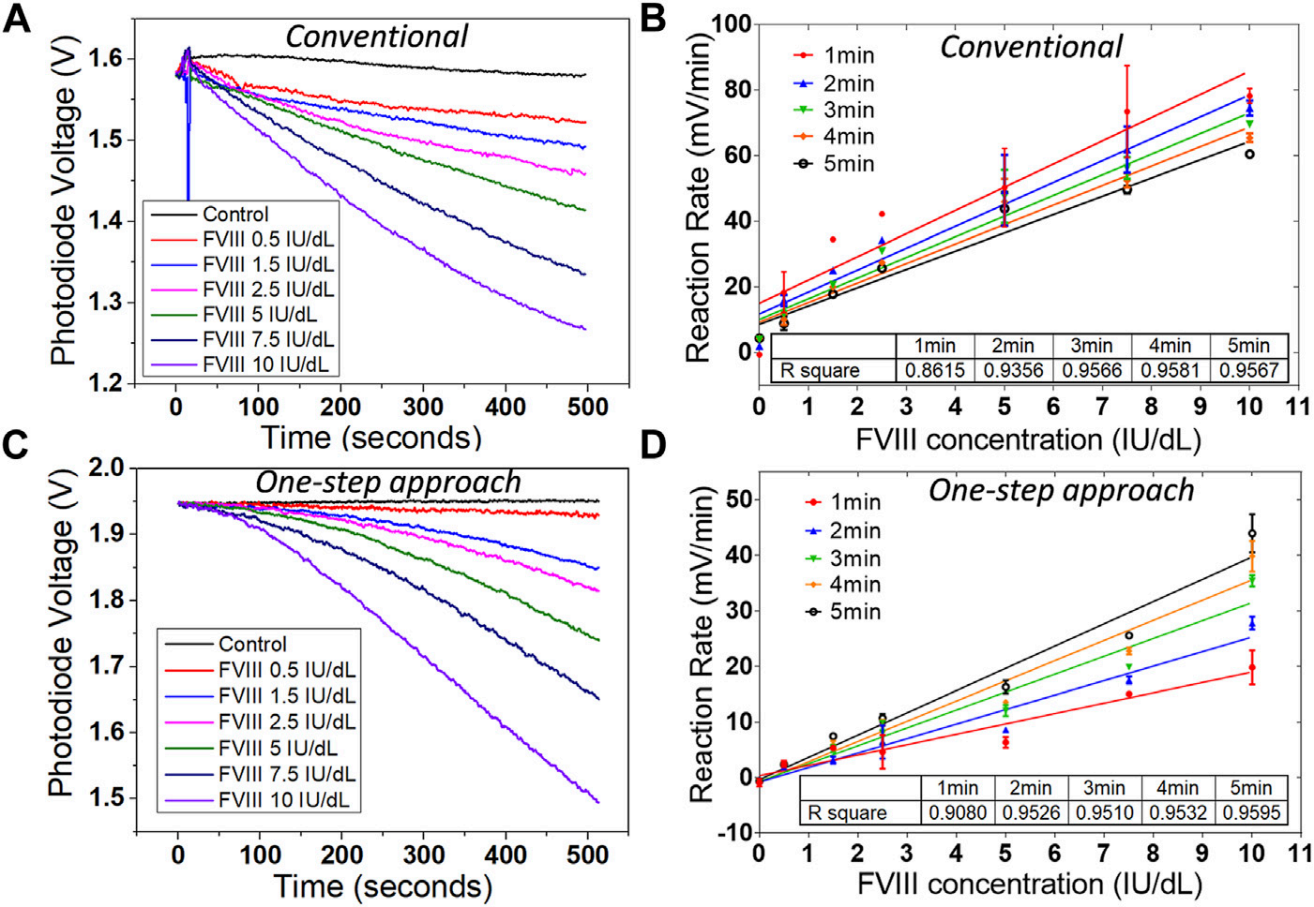
Results from diluted plasma on the sensing platform. **(A)** Reaction curves, and **(B)** reaction rates from six concentrations of diluted plasma samples on the sensing platform with conventional chromogenic method. **(C)** Reaction curves, and **(D)** reaction rates from six concentrations of diluted plasma samples on the sensing platform with the one-step chromogenic approach. Data are the mean ± SEM with three repeated measurements.

As mentioned in the previous section the plasma sample in a conventional chromogenic assay is required to be present in the first step to initiate the assay. This is not suitable for a point-of-care diagnostic device that requires the sample to be introduced as the final, and only, user step. Modifications to the protocol were therefore implemented to make it possible to add the plasma sample as the final step on our sensing platform. A conventional assay (Figure 1A) was undertaken on the sensing platform to validate the performance of the platform (Figure 4A). Instead of measuring the end-point value (Figure 2A), reaction rate is recorded (Figure 4B) to provide additional information of the reaction. The Factor VIII plasma calibrator (includes 100 IU/dl Factor VIII) was diluted with the reaction buffer to get six different concentrations of plasma samples: 0.5, 1.5, 2.5, 5, 7.5, and 10 IU/dl. The protocol for the conventional assay was used off the chip with all the incubation steps until the chromogenic substrate step for all the six concentrations of plasma samples. The solution was then added to the sensing platform and the recording started. The reaction was triggered by adding the substrate. Typical enzymatic reaction curves were obtained (Figure 4A) and the reaction rate for the first 1–5 min were calculated, which shown good linearity (Figure 4B).

The same six different concentrations of diluted plasma samples were also measured using the one-step chromogenic approach on the sensing platform (Figures 4C,D). All the reagents required for the assay except the plasma (Factor VIII) was mixed and placed on the chip surface of the sensing platform prior to the measurement at room temperature. The introduction of the plasma sample initiated the reaction, and the reaction rates were also calculated for the first 1–5 min (Figure 4D). The linearity of the reaction rate improved after the first 1 min and started to reach the plateau after 3 min. This indicates the operation time on the sensing platform can be as short as 3 min.

**FIGURE 5:**
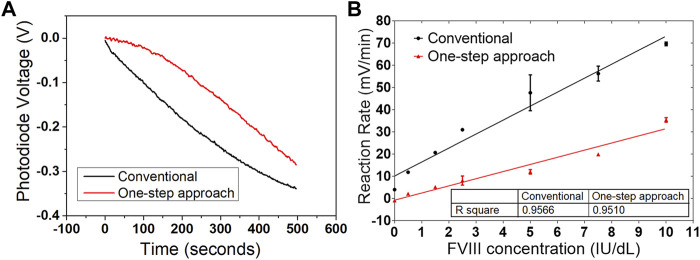
Comparison of the two chromogenic approaches on the sensing platform. **(A)** Reaction curves from both conventional and one-step approaches with diluted plasma containing 10 IU/dl Factor VIII. The difference on the waveform is obvious, but reasonable. **(B)** Reaction rates from both conventional and one-step approaches. The reaction rate from the one-step approach is slower, but with comparable linearity and sensitivity. Data are the mean ± SEM with three repeated measurements.

Although both assays showed a good linearity and sensitivity, there was a clear difference between the conventional method and the one-step approach (Figure 5). The raw signal from both diluted plasma samples with 10 IU/dl Factor VIII are plotted in Figure 5A for both methods. The magnitude of the signal change is similar, but there is a clear difference in the shape of the measured curve as a function of time. This is to be expected since for the conventional method, by the time the chromogenic substrate was added, the coagulation reactions among the clotting factors had already proceeded for more than 5 min. In the one-step approach, the reaction did not start until the plasma sample containing Factor VIII was added. There was therefore a delay before the chromogenic reaction started with the one-step approach. This is clearer on the reaction rate plot shown below in Figure 5B. The reaction rate from the one-step approach was around half the value for that from the conventional approach. However, this should not affect the performance of the one-step approach, since the sensitivity and detection range are still good enough for diagnostic purposes with haemophilia patients. The limit of detection obtained from the diluted plasma is around 0.27 IU/dl for the conventional method and 0.16 IU/dl for the one-step approach.

### Result from undiluted plasma samples

**FIGURE 6:**
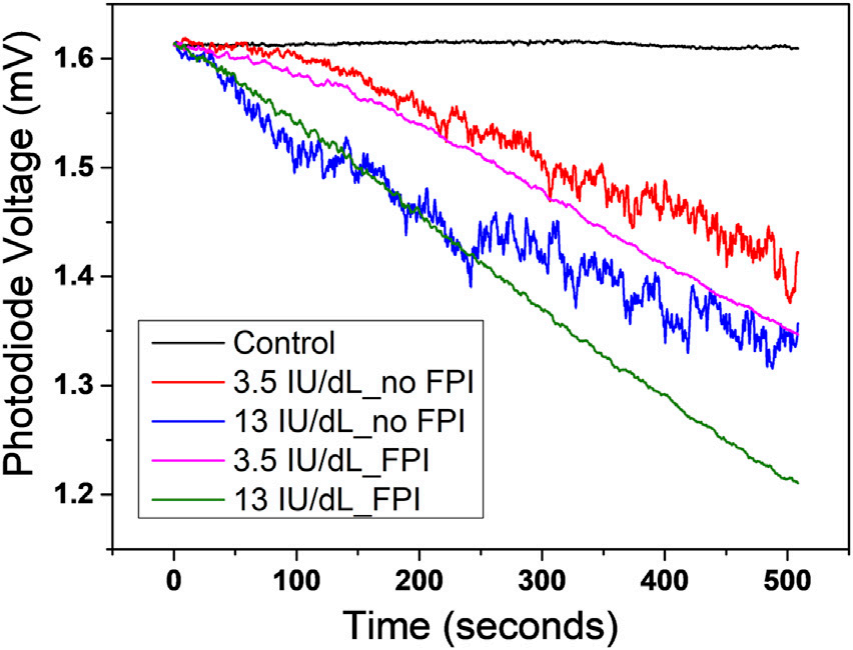
Comparison of the reaction curves with and without the addition of fibrin polymerization inhibitor (FPI). The chromogenic assay with undiluted plasma produces noisy signals (red and blue curves in the figure) owing to the generation of cross-linked fibrin. Experiments confirmed that the addition of FPI dramatically reduced the noise level and at the same time did not affect the assay reaction (magenta and green curves in the figure).

The dilution of the plasma sample is time-consuming and requires a skilled operator. However, the chromogenic assay with undiluted plasma produces noisy signals due to the generation of cross-linked fibrin (Figure 6). To overcome this issue, in the present case undiluted plasma samples were measured with modifications to the protocol, which included the addition of high concentration of fibrin polymerization inhibitor (FPI) Gly-Pro-Arg-Pro amide (30 mg/ml) and a reduction of the reagent and sample volume (from 300 to 50 µL). Experiments confirmed that the addition of FPI did not affect the assay reaction (Figure 6), but in the meanwhile, we observed that there was a dramatical reduction in the signal noise level and an improvement in the stability of the measurement.

**FIGURE 7:**
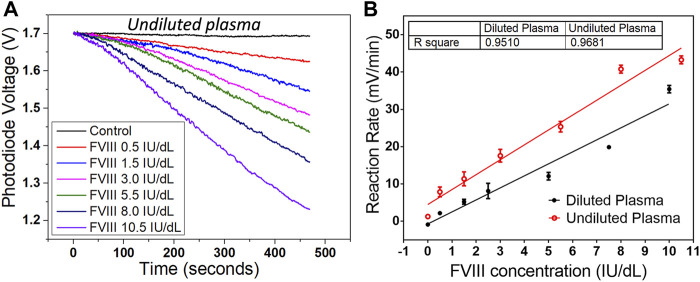
Result from undiluted plasma on the sensing platform. **(A)** Reaction curves from six concentrations of undiluted plasma samples on the sensing platform with one-step chromogenic approach. **(B)** Comparison of the reaction rates between the diluted and undiluted plasma for the first 3 min. The undiluted plasma shows a slightly faster reaction rate and a better linearity. Data are the mean ± SEM with three repeated measurements.

The Factor VIII deficient plasma was spiked with the Factor VIII plasma calibrator (includes 100 IU/dl Factor VIII) to mimic haemophilia patient plasma samples. Ideally, there should no Factor VIII inside the Factor VIII deficient plasma, but in reality, there is always a very low concentration of Factor VIII. The depleted Factor VIII used in this work contained 0.5 IU/dl Factor VIII, introducing only a very small error in the experiments. Six plasma samples, to mimic undiluted plasma, with concentrations of Factor VIII of 0.5, 1.5, 3.0, 5.5, 8.0, 10.5 IU/dl, were made up. The same one-step measurement method introduced in the previous section was used with the six concentrations of plasma. The results obtained were comparable to those for the assay system that relied on pre-dilution of the plasma to lower concentrations (Figure 7). The limit of detection is 0.16 IU/dl with the diluted plasma and 0.21 IU/dl with the undiluted plasma. The comparison between the conventional and one-step chromogenic approaches in terms of testing time, incubation step, testing temperature, sample volume, detecting range, limit of detection, sample type, and operator is listed in Table 1.

**TABLE 1:** Comparison of the conventional and one-step approach.

	*Conventional Method*	*One-step Approach*
Time	>20 min	<5 min
Incubation	Yes	No
Temperature	37°C	Room temperature (21°C)
Volume	300–600 µL	<50 µL
Range	0.5–200 IU/dl	0.5–20 IU/dl
Limit of Detection	0.3 IU/dl	0.2 IU/dl
Sample	Diluted plasma	Whole blood plasma
Manipulation	Skilled person	Non-skilled person

## Conclusion

We have successfully demonstrated an innovated one-step chromogenic approach for rapid measurement of the Factor VIII activity levels with a low-cost digital mobile sensing platform for haemophilia A. The new system uses a novel one-step chromogenic assay suitable for implementation in a stand-alone hand-held point-of-care format. The system is underpinned by the widely used conventional chromogenic assay, but with modifications. The new assay method has many advantages over the conventional assay: no dilution or defibrination of the sample is required, no incubation steps are involved, assay time is much less, assay volume is reduced, and minimal training is required for the user. Our results show comparable performance to the gold standard method, which makes our sensing platform a potential candidate for Factor VIII companion diagnosis by haemophilia A patients at home.

## Data Availability

The datasets for this study can be found in the University of Glasgow library: http://dx.doi.org/10.5525/gla.researchdata.1242.
